# Publication of Masatoshi Nei’s Memoir “*My Life as a Molecular Evolutionist*”

**DOI:** 10.1093/molbev/msab058

**Published:** 2021-04-10

Molecular Biology and Evolution (MBE) is pleased to announcethat Masatoshi Nei recently published a memoir entitled “*My Life as a Molecular Evolutionist*” from the Institute of Genomics and Evolutionary Medicine, Temple University Philadelphia, PA. The purpose of this memoir is to discuss the origin and development of the discipline of molecular evolution from the point of view of a cofounder of *MBE*. He was born in Japan in 1931 and immigrated to the United States in 1969 to do the work on molecular evolution. Since then, most of his professional work was done in the United States. In this memoir, he discusses how he grew up in Japan and how he ended up with working on molecular evolution, including some of enjoyment and grief in conducting the work. He emphasizes the importance of working with collaborators and indicates that many of his studies were done in these collaborations. The memoir is about 200 pages long and consists of 9 chapters and additional sections (see Box 1).


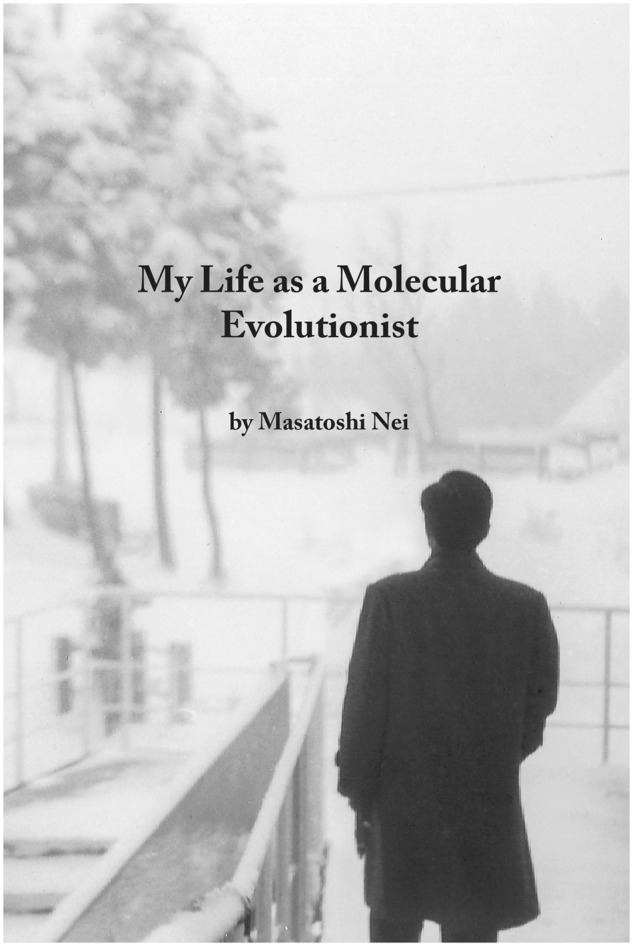

Box 1. **Contents** **of Nei memoir**

*Preface*

Chapter 1.*  Introduction*

      Chronological Timeline

Chapter 2.  *My Childhood*

Chapter 3.  *University Education*

      University of Miyazaki

      Kyoto University

Chapter 4.  *Studies in the USA*

      University of California at Davis

      North Carolina State University

Chapter 5.  *National Institute of Radiological Sciences in Chiba, Japan*

      Marriage and Children

      Kimura’s Neutral Theory of Molecular Evolution

      Use of Computers in Genetics

      International Congress of Genetics in Tokyo and Kyoto

Chapter 6.  *Brown University in the USA*

      Adapting to the American Way of Life

      Theory of Genetic Distance

Chapter 7.  *University of Texas Health Science Center at Houston*

      Neutral Theory of Molecular Evolution *vs.* The Theory of Mutation-Driven Evolution

      Gene Genealogy, Pseudogenes, and Neutral theory

      Human Evolution

      Meetings in Texas

      Visit to India: A Mysterious Country

      Moscow and Other European Cities

      China and Sweden

      Titisee Meeting in Germany

      Natural Selection at the Major Histocompatibility (MHC) Loci

      Book on *Molecular Population Genetics and Evolution*

      Neighbor-Joining Method of constructing Phylogenetic Trees

      Gordon Research Conferences

      Founding a New Journal called “*Molecular Biology and Evolution (MBE)*”

      International and Domestic Meetings

Chapter 8.  *Pennsylvania State University*

      Work on Committees

      Molecular Evolution and Phylogenetic Trees

      Australia: a Continent of Isolation

      Origins of the Japanese Population

     Official Start of *the Society for Molecular Biology and Evolution (SMBE)*

      MEGA: Molecular Evolutionary Genetics Analysis Software

      Molecular Evolution and Phylogenetic Trees

      Australia: a Continent of Isolation

      Origins of the Japanese Population

      Trip to Northern Greece and Hungary

      Sabbatical leave in Japan, 1997

     Penn State—SMBE Symposium on Molecular and Genomic Evolution

      Another trip to China

      Meeting with the emperor and other recognitions

Chapter 9.  *Temple University*

      A Mild Form of Stroke and Temple University

      Symposium on Molecular Evolution at Temple University

      My Work at Temple University

*Epilogue and Acknowledgements*

*References*

*Index*

Masatoshi Nei’s studies of evolutionary problems started from an investigation on the origin of genetic variation in populations, but as genetics has grown up into molecular biology, he has also become a molecular evolutionist. Working alone or with his students and collaborators, he continuously developed various new concepts of evolution and new statistical methods. Early on in these studies, he understood the importance of using computers in biology, bioinformatics, and genomics. Because of these researches, his work is widely appreciated. For those who are interested, the memoir is now available for purchase at the following website: https://*press.arctangent.com.*

